# Determination of Melting Parameters of Cyclodextrins Using Fast Scanning Calorimetry

**DOI:** 10.3390/ijms232113120

**Published:** 2022-10-28

**Authors:** Askar K. Gatiatulin, Ivan A. Grishin, Aleksey V. Buzyurov, Timur A. Mukhametzyanov, Marat A. Ziganshin, Valery V. Gorbatchuk

**Affiliations:** A.M. Butlerov Institute of Chemistry, Kazan Federal University, 18 Kremlevskaya, 420008 Kazan, Russia

**Keywords:** cyclodextrin, fusion, melting, thermal analysis, fast scanning calorimetry, chip calorimetry

## Abstract

The first evidence of native cyclodextrins fusion was registered using fast scanning calorimetry (FSC) with heating rates up to 40,000 K s^−1^. The endothermal effects, detected at low heating rates, correspond to the decomposition processes. Upon the increase of the heating rate the onset of these effects shifts to higher temperatures, reaching a limiting value at high heating rates. The limiting temperatures were identified as the melting points of α-, β- and γ-cyclodextrins, as the decomposition processes are suppressed at high heating rates. For γ-cyclodextrin the fusion enthalpy was measured. The activation energies of thermal decomposition of cyclodextrins were determined by dependence of the observed thermal effects on heating rates from 4 K min^−1^ in conventional differential scanning calorimetry to 40,000 K s^−1^ in FSC. The lower thermal stability and activation energy of decomposition of β-cyclodextrin than for the other two cyclodextrins were found, which may be explained by preliminary phase transition and chemical reaction without mass loss. The obtained values of fusion parameters of cyclodextrins are needed in theoretical models widely used for prediction of solubility and solution rates and in preparation of cyclodextrin inclusion compounds involving heating.

## 1. Introduction

Native cyclodextrins (CDs) are pharmaceutically important biomolecules, which applications require a knowledge of their melting parameters. Melting points and fusion enthalpies are essential parameters used in theoretical models for prediction of solubility [1,2,3] and in correlations with dissolution rates [4,5], which are key physical values for pharmaceuticals [6,7]. CDs are used as excipients enhancing the solubility and dissolution rate of active pharmaceutical ingredients [8,9]. So, the melting parameters of CDs may be used to expand the existing predictive approaches also to the solubility and dissolution rates of CDs and their inclusion compounds. The problem is in a limited thermal stability of CDs, which does not allow to study their fusion because CDs decompose long before melting point when studied by conventional methods, e.g., by differential scanning calorimetry (DSC) [10]. So, a special experimental technique is necessary to overcome this problem.

A state-of-the-art method for determination of melting parameters for compounds of low thermal stability is fast scanning calorimetry (FSC) [6,11,12,13]. FSC was used to determine melting properties of thermally labile biomolecules: proteins [14], peptides [15], amino acids [5], pharmaceuticals [16,17], mono and disaccharides [1], and nucleobases [18]. This method uses special chip sensors that are both sample holders and electrical circuits for sample heating and determination of its temperature and heat flow at scanning rates of several thousand K per second due to the extremely low addenda heat capacity and sample size [19]. Due to the rapid heating, the fusion may complete before the decomposition processes of studied compounds [15,18]. Even fastest conventional DSC (Hyper-DSC by Perkin Elmer) used to detect fusion of pharmaceuticals within separate pans [20] with heating rates of a few hundred K min^−1^ is not suitable to study the melting of cyclodextrins because they have too big and thermally labile molecules. So, in the present work, FSC method was first used to determine melting parameters of native cyclodextrins.

Our approach to the study of CDs melting properties is based on the analysis of their thermal effects dependence on heating rate. In relatively slow thermal analysis, decomposition of cyclodextrins occurs [10]. This process for β-cyclodextrin and other carbohydrates has a significant dependence of decomposition temperature on heating rate [21]. So, conventional DSC method was used in the present work to determine kinetic parameters of thermal decomposition for three native CDs: α-, β-, and γ-cyclodextrin. From these data, the correlation of decomposition temperature with heating rate was estimated. FSC method was applied to find the scanning rate above which this correlation breaks resulting in relatively small further shift of the observed endothermic effect by temperature. Such marginal dependence on heating rate is intrinsic to fusion of biomolecules in FSC studies [1,15,18]. From the onset temperature of main thermal effect in this range of heating rates the melting points of native CDs were determined.

## 2. Results and Discussion

**Figure 1:**
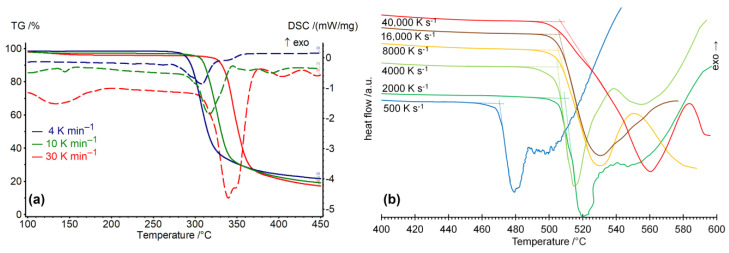
The influence of heating rates on the curves of thermal analysis of dry α-cyclodextrin: (**a**) simultaneous TG (solid lines) and DSC (dashed lines), (**b**) FSC experiment, where the cross-points of dotted lines indicate the onset points *T*_o_ of the main endothermic effect of decomposition or fusion.

To study the kinetics of thermal decomposition and fusion of native CDs, the samples of αCD, βCD, and γCD were studied by methods of simultaneous TG/DSC with heating rates of 4, 10 and 30 K min^−1^ and FSC with the rates of 30–40,000 K s^−1^. The TG/DSC and FSC curves obtained are shown in Figure 1 for αCD and in Supplementary Information (SI) for βCD and γCD. From these curves the onset temperatures *T*_o_ of the main endothermic peaks were determined for each sample, Table 1.

**Figure 2:**
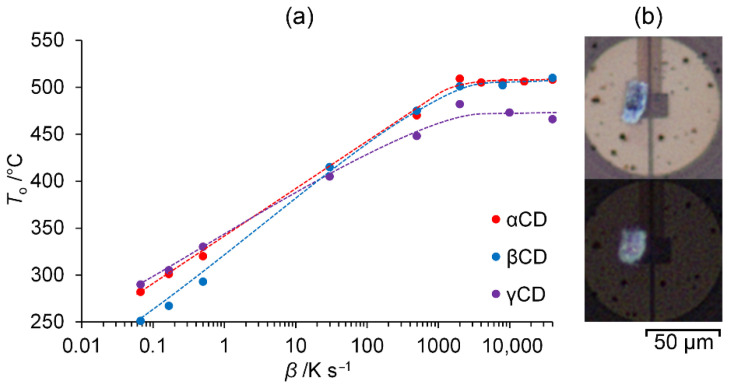
(**a**) Correlation of onset temperatures (*T*_o_) of main endothermic peak with the heating rate *β* in DSC and FSC experiments for dry native cyclodextrins (the lines are given to guide the eye); (**b**) the microscope pictures of αCD sample in polarized light before (**upper**) and after (**lower**) melting by heating to 520 °C and cooling to room temperature with the rate of 40,000 K s^−1^.

The analysis of data obtained indicates that *T*_o_ values significantly increase with the increase in heating rate up to 2000 K s^−1^, Figure 2a. At higher heating rates *β* of 2000–40,000 K s^−1^, *T*_o_ values do not change significantly, Figure 2a. Such behavior can be explained by two different processes corresponding to *T*_o_. At low heating rates, the values *T*_o_ correspond to the onset points of thermal decomposition. Since this decomposition is a chemical process, *T*_o_ increases significantly with increasing heating rate. At higher heating rates, the nearly constant *T*_o_ values indicate that a fusion occurs first in cyclodextrins, preceding their further decomposition. Thus, for α-, β-, and γ-cyclodextrins, the melting point *T*_m_ is 507, 501, and 474 °C, respectively. These values are calculated as the average ones for heating rates 2000–40,000 K s^−1^.

Melting of αCD was revealed also by polarized light microscopy. Upon heating above its melting point of 520 °C derived from *T*_o_ vs. *β* correlation and cooling to room temperature at a rate of 40,000 K s^−1^, the sample of αCD contracts to more round shape remaining colorless and crystalline, Figure 2b, which shows that α-cyclodextrin melts without decomposition at its *T*_m_ value found.

The observed differences in melting points of native CDs are consistent with thermodynamic properties of anhydrous CDs. The anhydrous γCD has much more negative enthalpy of solution in water and N,N-dimethylformamide, compared to αCD and βCD [22]. Presuming that all CDs have a similar state in solution, the higher energy of solid γCD can explain its lower melting point.

**Figure 3:**
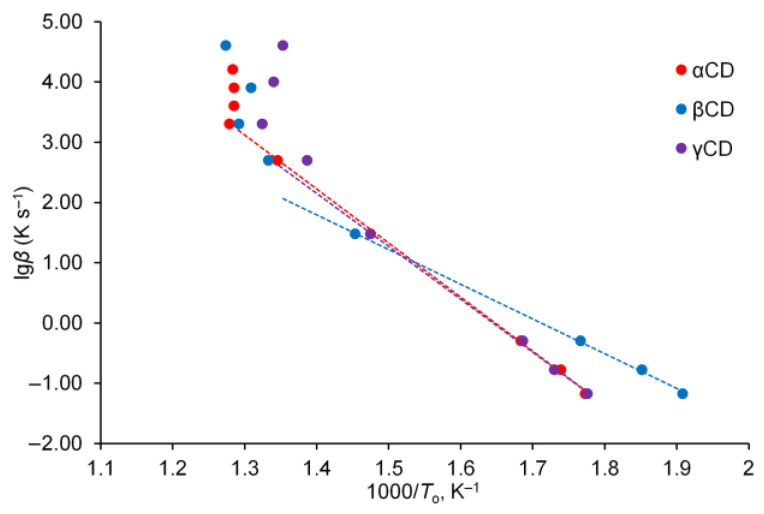
Fitting by Flynn–Wall–Ozawa method for dependence of *T*_o_ on heating rate.

To calculate the kinetic parameters of CDs decomposition, slope of the plot ‘lg*β* vs. 1000/*T*_o_’ was determined for each studied CD, Figure 3. This correlation is linear for DSC and FSC data for heating rates below 2000 K s^−1^ for αCD, and below 500 K s^−1^ for βCD and γCD. According to the Flynn–Wall–Ozawa method [21], its slope gives activation energy *E*_a_ of the initial stage of thermal decomposition (SI). The activation energy of this process is equal to 163, 105, and 160 kJ mol^−1^ for αCD, βCD, and γCD, respectively.

The TG/DSC data obtained also allow to estimate *E*_a_ value from the thermogravimetric data in the wide range of decomposition degree. Approximation of these thermokinetic data by model methods shows that for the native CDs studied the most appropriate kinetic model is CnB which corresponds to the *n*th order reaction with autocatalysis, the corresponding conversion function is *f*(*α*) = (1 − *α*)*^n^* · (1 + *K*_cat_*α*). In this case, the optimal activation energies *E*_a_ are 147, 177, and 156 kJ mol^−1^, respectively.

The TG data were also analyzed using isoconversional model-free methods: differential Friedman and integral Flynn–Wall–Ozawa. These methods do not require any assumptions of the kinetic equation other than the Arrhenius-type temperature dependence of the reaction rate [23]. In the range of conversion degrees 0.1–0.7, these methods give average *E*_a_, respectively, 149 and 146 kJ mol^−1^ for αCD, 169 and 174 kJ mol^−1^ for βCD, 154 and 156 kJ mol^−1^ for γCD (SI). These *E*_a_ values are nearly the same and, in the case of αCD and γCD, are close to the *E*_a_ values calculated from DSC and FSC data for the initial stage of decomposition.

The activation energies calculated from calorimetric and TG data are close for αCD and γCD —the difference does not exceed 10–15 kJ mol^−1^. For βCD, the difference between activation energies calculated by different methods is significant: 65–73 kJ mol^−1^. Such difference can be explained by the different onset temperatures observed in TG and DSC measurement. For βCD, the average difference between TG and DSC onset points is 33 K, while as for αCD and γCD it is equal to 12 and 6 K, respectively. For βCD, earlier DSC onset can be explained by preliminary phase transition or intramolecular reaction without a change in mass. Moreover, for βCD, a phase transition is observed at 222 °C (SI), which has been observed also elsewhere [10]. All these effects can increase the energy of βCD thus decreasing activation energy of initial step of its main thermal degradation.

**Figure 4:**
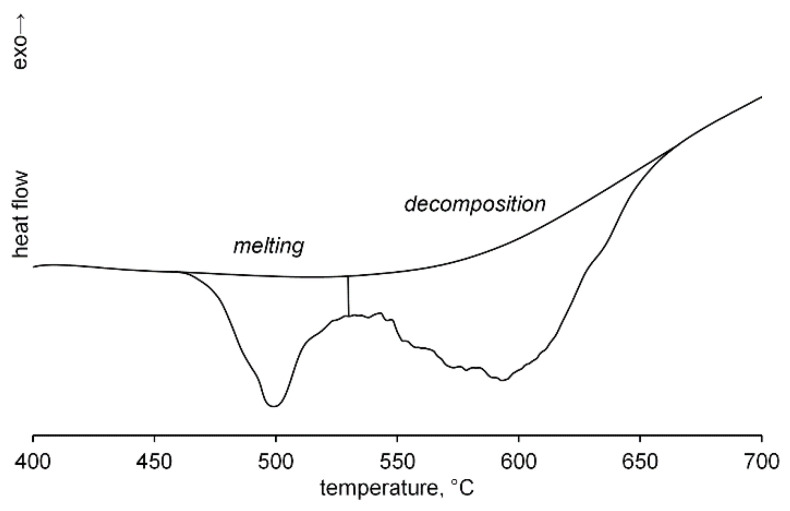
FSC curve for γ-cyclodextrin at heating rate of 10,000 K s^−1^.

The FSC experiment for γCD allows determination of its fusion enthalpy Δ*H*_m_. This cyclodextrin has the lower melting point than the other CDs studied, Table 1, and its melting and decomposition peaks are separate at the heating rate of 10,000 K s^−1^, Figure 4. So, the molar fusion enthalpy Δ*H*_m_ of γCD can be calculated. For this study, we additionally determined the molar heat capacity *C*_p,m_ of this cyclodextrin in the range of 80–220 °C (SI), which is needed to find its sample mass in FSC experiment. The temperature dependence of this parameter is expressed by the equation *C*_p,m_ (J mol^−1^ K^−1^) = 781 + 9.13*T* − 0.0043*T*². At 298 K, this equation gives the value of *C*_p,m_ = 1556 J mol^−1^ K^−1^, which is in a good agreement with the previously determined value of *C*_p,m_ = 1568 J mol^−1^ K^−1^ [24]. Two runs of FSC experiments for γCD at a rate of 10,000 K s^−1^ give molar fusion enthalpy of Δ*H*_m_ = 221 ± 9 kJ mol^−1^ (SI). The corresponding molar fusion entropy of γCD (at *T*_m_ = 474 °C) is Δ*S*_m_ = 296 ± 12 J mol^−1^ K^−1^.

## 3. Materials and Methods

### 3.1. Materials

α-Cyclodextrin and γ-cyclodextrin were obtained commercially from Sigma-Aldrich with Cat. Nos. 28705, 779431, respectively. β-Cyclodextrin was obtained from ICN, Cat. No. 190053.

### 3.2. Simultaneous TG/DSC Experiment

Before the experiment, all CD samples were dried at 140 °C and 100 Pa in a vacuum oven for 8 h. The residual hydration of CD samples was no more than 2% wt. or 1.1 mol water per mol CD according to TG data.

The device of simultaneous thermogravimetry and differential scanning calorimetry (TG/DSC) Netzsch STA 449 C Jupiter was used to determine thermal decomposition curves of dried cyclodextrins with heating rates of 4, 10, and 30 K min^−1^ in an argon flow of 75 mL min^−1^. In this experiment, the CD samples (10–15 mg) were studied in aluminum crucibles (40 μL) with lids having 3 holes of 0.5 mm in diameter. Before heating, the samples were purged with argon at room temperature inside the device until the constant weight.

### 3.3. Model-Free Methods of Thermokinetic Analysis

Two model-free methods of Friedman [25] and Ozawa–Flynn–Wall [26,27] were used for approximation of the experimental data according to the recommendation of International Confederation for Thermal Analysis and Calorimetry [28].

In Friedman method, for a linear non-isothermal program, the next equation is used:

ln [*β_i_*·(*dα*/*dT*)*_α,i_*] = ln *A* + ln *f*(*α*) − *E*_a_/*RT_α,i_*

where *E*_a_ is activation energy, *β* is heating rate, *i* marks an individual heating rate, *A* is pre-exponential factor and *α* is the extent of conversion. The *E*_a_ and pre-exponential factor are calculated from the slope of the plots of ln (*dα*/*dT*) vs. 1/*T*.

The Flynn−Wall−Ozawa method is a model-free method that requires the detection of temperatures corresponding to isoconversional values of α from experiments at different heating rates *β* [23]:

ln *β* = 5.523 − 1.052(*E*_a_/*RT*)

Thus, the plot of ln *β* vs 1/*T* gives straight line with slope −1.052(*E*_a_/*R)*.

### 3.4. DSC Experiment

The molar heat capacity *C*_p,m_ of dry γ-cyclodextrin was measured using Netzsch DSC204 F1 Phoenix differential scanning calorimeter. This procedure using sapphire disk as a standard sample was described in detail earlier [12].

### 3.5. Fast Scanning Calorimetry

FSC experiments were performed using Flash DSC2+ (Mettler Toledo, Switzerland) with MultiSTAR UFH1 sensors. Before use, each UFH1 sensor was conditioned and corrected according to the procedure defined by the manufacturer. The measured temperature of the sensors was calibrated using organic compounds having well known melting temperatures as described elsewhere [29]. In FSC experiment, crystal aggregates with a total mass of 20–50 ng were placed at the center of sensor. Temperature scan of these samples was performed in argon dynamic atmosphere with 80 ml/min flow rate.

To get rid of the dehydration thermal effect, which was observed elsewhere at temperatures up to 160 °C [30,31,32], all CD samples were preheated directly on FSC chip to a temperature 200 °C in argon flow. Such heating was repeated several times until the heat flow in FSC curves reached a constant level.

Activation energies of initial stage of CDs decomposition were calculated by Flynn–Wall–Ozawa method described in detail for other carbohydrate biopolymers [21].

The molar fusion enthalpy of γCD was determined using FSC as described elsewhere [11]. In this experiment, the sample of γCD was cyclically heated and cooled 5 times in the range of 70–200 °C before melting. The heating and cooling curves in these runs and the molar heat capacity *C*_p,m_ of γCD from the DSC experiment were used to calculate the mass of sample on FSC chip. The molar enthalpy of melting was calculated from the thermal effect in FSC curve for the same sample heated up to 700 °C.

## 4. Conclusions

The melting points of thermally unstable α-, β-, and γ-cyclodextrin and the kinetic parameters of their thermal decomposition were determined using fast scanning calorimetry, which gives a possibility to shift decomposition temperature of thermally unstable compound above its fusion process using very fast heating rates. The same approach may be extended to crystalline inclusion complexes of native cyclodextrins with various medical drugs. β-Cyclodextrin was found to have a lower activation energy of decomposition what may be explained by preliminary phase transition and the following chemical reaction without loss of mass. Such specific feature of β-cyclodextrin thermal stability must be considered during preparation of its complexes involving heating.

The determined fusion enthalpy and entropy of γ-cyclodextrin may be used to check the theoretical models predicting these properties for organic compounds from structural parameters of their molecules. Melting points and enthalpy obtained in this work are needed for models predicting aqueous solubility of CDs which can be helpful for their applications as excipients enhancing the solubility and dissolution rate of active pharmaceutical ingredients.

## Figures and Tables

**Table 1:** Onset temperatures (*T*_o_) of main endothermic peak for anhydrous natural cyclodextrins at different heating rates (*β*) *.

α-Cyclodextrin		β-Cyclodextrin		γ-Cyclodextrin	
*β*/K s^−1^	*T*_o_/°C	*β*/K s^−1^	*T*_o_/°C	*β*/K s^−1^	*T*_o_/°C
0.0667	291	0.0667	251	0.0667	290
0.1667	302	0.1667	267	0.1667	305
0.5	321	0.5	293	0.5	320
500	470	30	415	30	405
2000	509	500	477	500	448
4000	505	2000	501	2000	482
8000	506	8000	491	10,000	473
16,000	506	40,000	512	40,000	466
40,000	508				

* Heating rates of 0.0667, 0.1667 and 0.5 K s^−1^ (4, 10 and 30 K min^−1^, respectively) were used in DSC experiments, 30–40,000 K s^−1^ in FSC experiments.

## Data Availability

Not applicable.

## Supplementary Materials

The following supporting information (FSC and TG/DSC curves, thermokinetic analysis, molar heat capacity and Δ*H*_m_ calculations for γ-cyclodextrin) can be downloaded at: https://www.mdpi.com/article/10.3390/ijms232113120/s1. Reference [21] is cited in the supplementary materials.
