# Supplementary material for: Determination of Melting Parameters of Cyclodextrins Using Fast Scanning Calorimetry
Source: Int J Mol Sci. 2022 Oct 28;23(21):13120. doi: 10.3390/ijms232113120 (PMC9655725; doi:10.3390/ijms232113120)
Supplement: Supplementary file 1 [file ijms-23-13120-s001.zip › ijms-1974505-supplementary.pdf]

Determination of Melting Parameters of Cyclodextrins Using Fast Scanning Calorimetry

Supplementary Material

Table of contents

FSC curves ... 2

TG/DSC curves ... 3

Correlation between onset temperatures obtained from DSC and TG data ... 4

Flynn-Wall-Ozawa analysis for onset points ... 4

Model-free thermokinetic analysis for CDs thermal decomposition (TG data) ... 5

Thermokinetic analysis — best fit model ... 8

Molar heat capacity of anhydrous γ-cyclodextrin ... 9

Data on γ-cyclodextrin melting ... 11

References ... 12

FSC curves

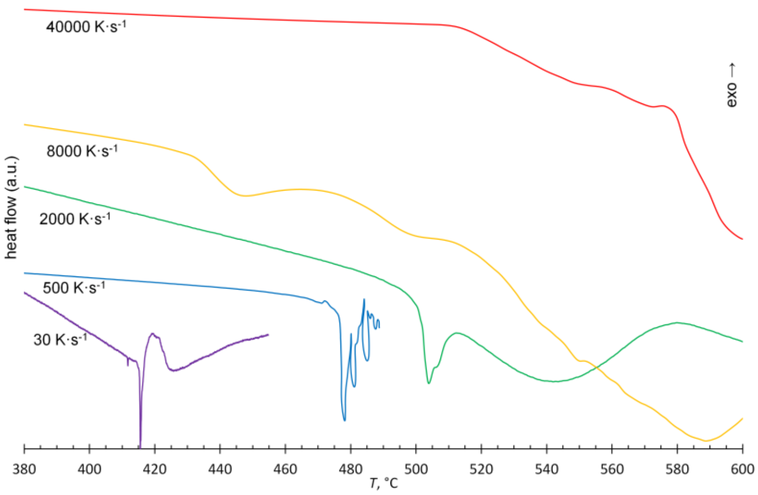

Figure S1. FSC curves for beta-cyclodextrin.

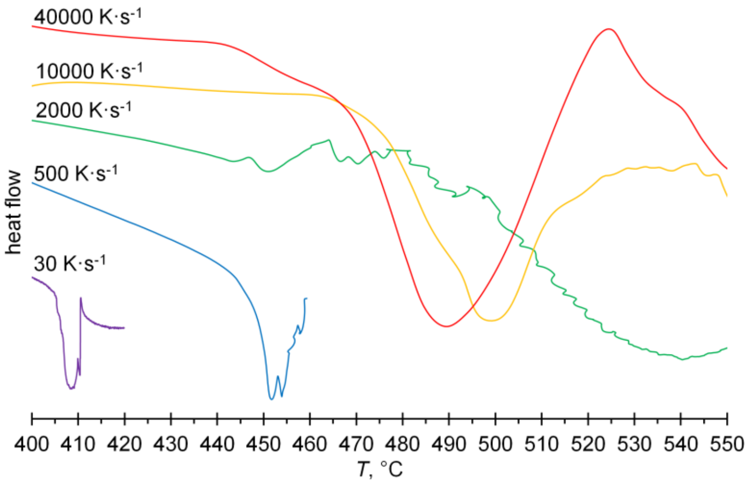

Figure S2. FSC curves for gamma-cyclodextrin.

TG/DSC curves

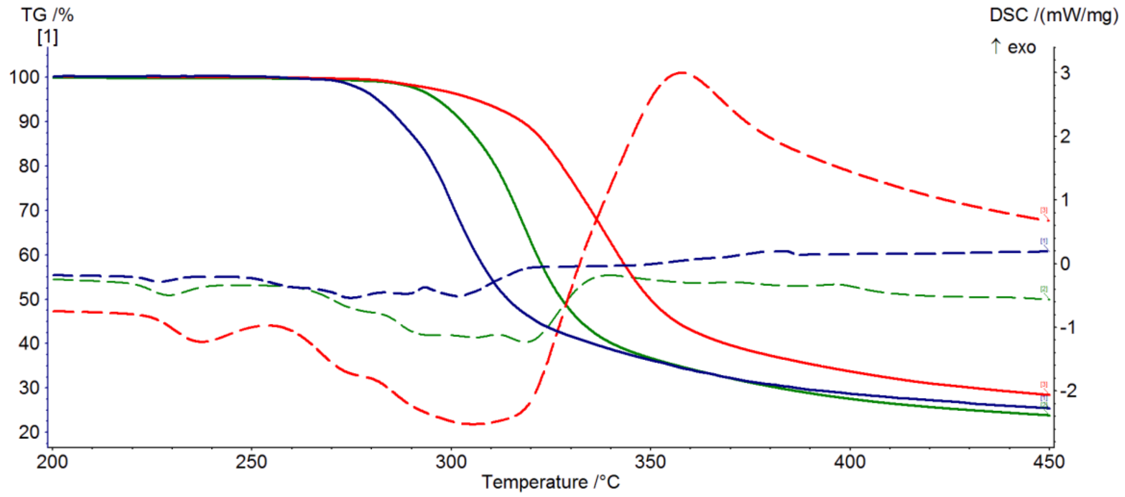

Figure S3. Combined TG/DSC analysis for beta-cyclodextrin at heating rates 4 (blue), 10 (green) and 30 (red) K·min^{-1}.

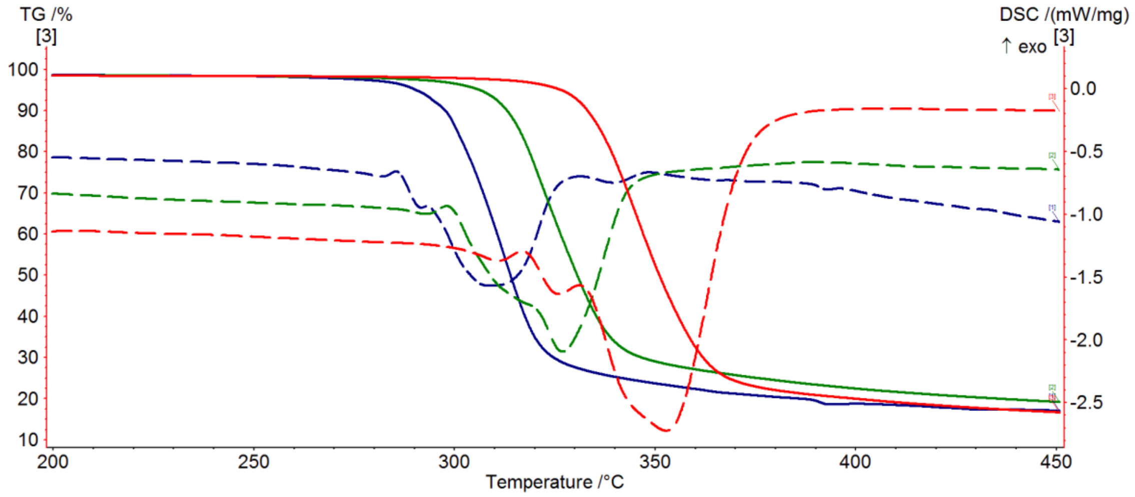

Figure S4. Combined TG/DSC analysis for gamma-cyclodextrin at heating rates 4 (blue), 10 (green) and 30 (red) K·min^{-1}.

Correlation between onset temperatures obtained from DSC and TG data

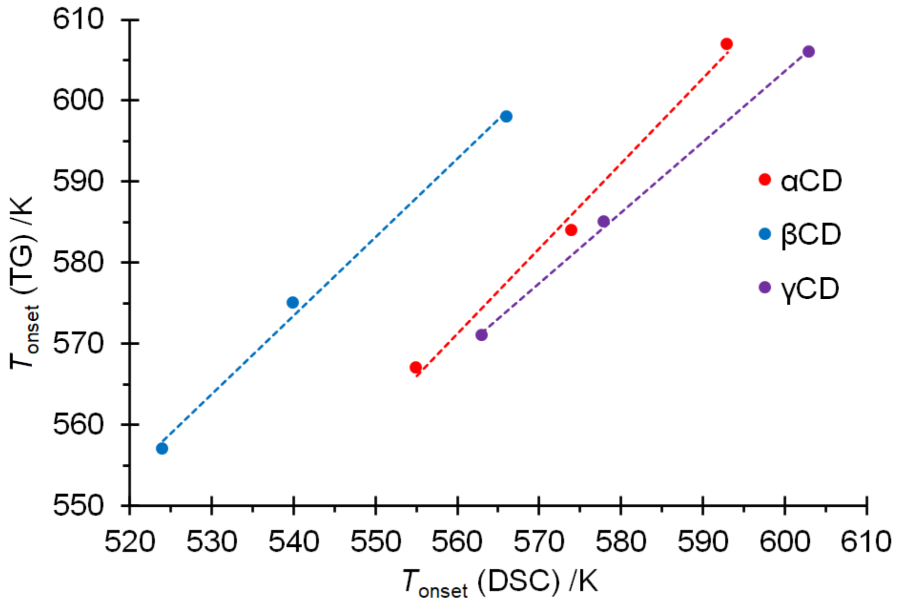

Figure S5. Correlation between onset temperatures of CDs decomposition obtained by TG and DSC.

Flynn-Wall-Ozawa analysis for onset points

In Flynn-Wall-Ozawa (FWO) method, T_o points (DSC and FSC) were plotted in coordinates lg heating rate (lgβ) against 1000/T_o. Linear dependencies corresponding to decomposition onset have a slope equal to −0.4567(E_a/R).[1]

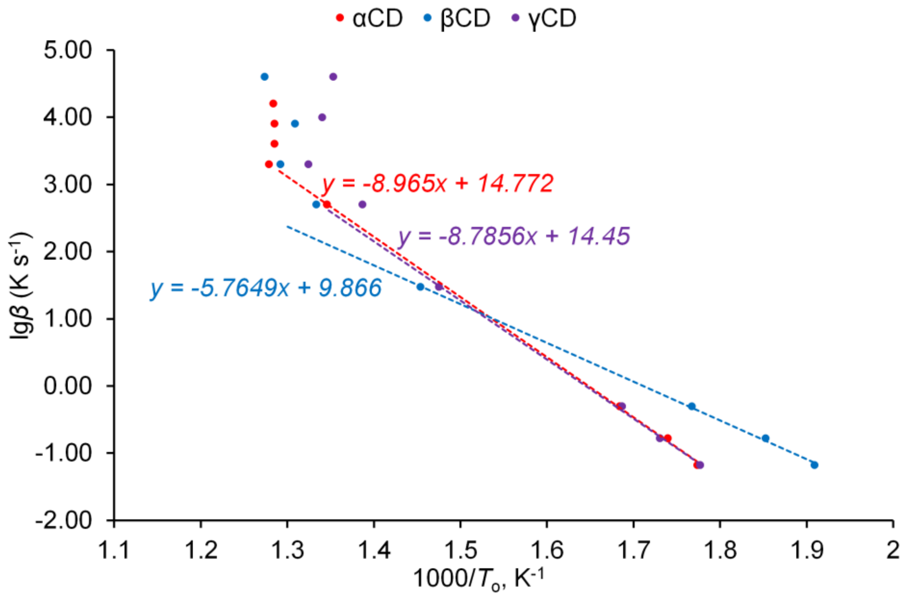

Figure S6. Flynn–Wall–Ozawa analysis for onset points of decomposition of natural cyclodextrins.

Corresponding E_a for initial stage of decomposition:

αCD: E_a = −8.97 · R / (−0.4567) = 163 (kJ mol^{-1})

βCD: E_a = −5.76 · R / (−0.4567) = 105 (kJ mol^{-1})

γCD: E_a = −8.79 · R / (−0.4567) = 160 (kJ mol^{-1})

Model-free thermokinetic analysis for CDs thermal decomposition (TG data)

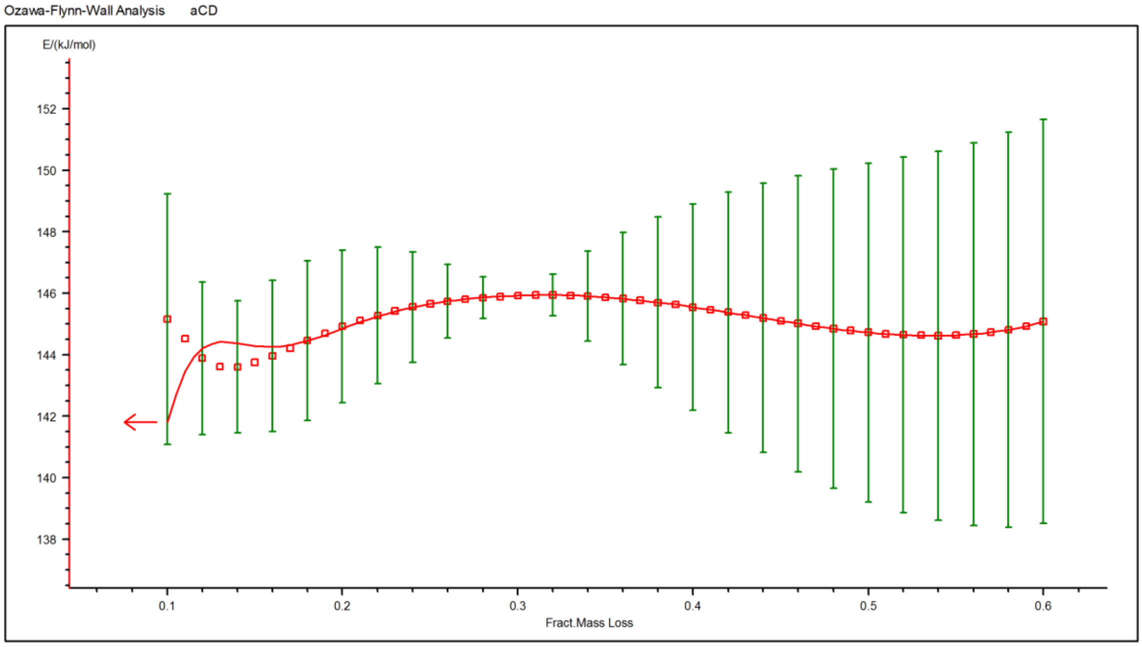

Figure S7. Isoconversional kinetic analysis (Flynn–Wall–Ozawa method) for alpha-cyclodextrin thermal decomposition.

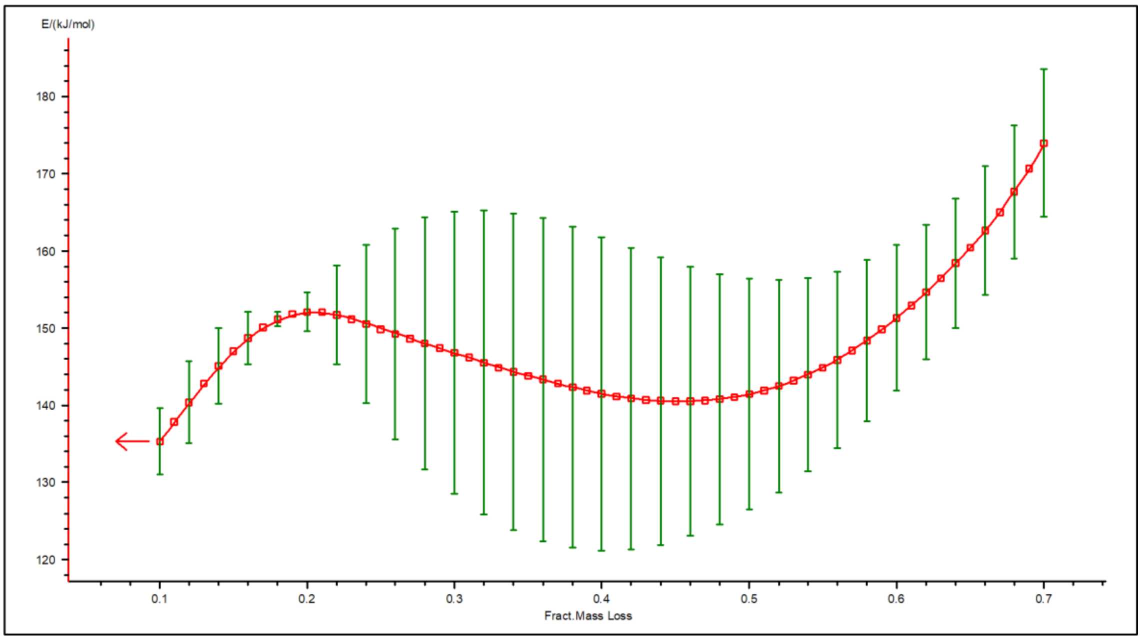

Figure S8. Isoconversional kinetic analysis (Friedman method) for alpha-cyclodextrin thermal decomposition.

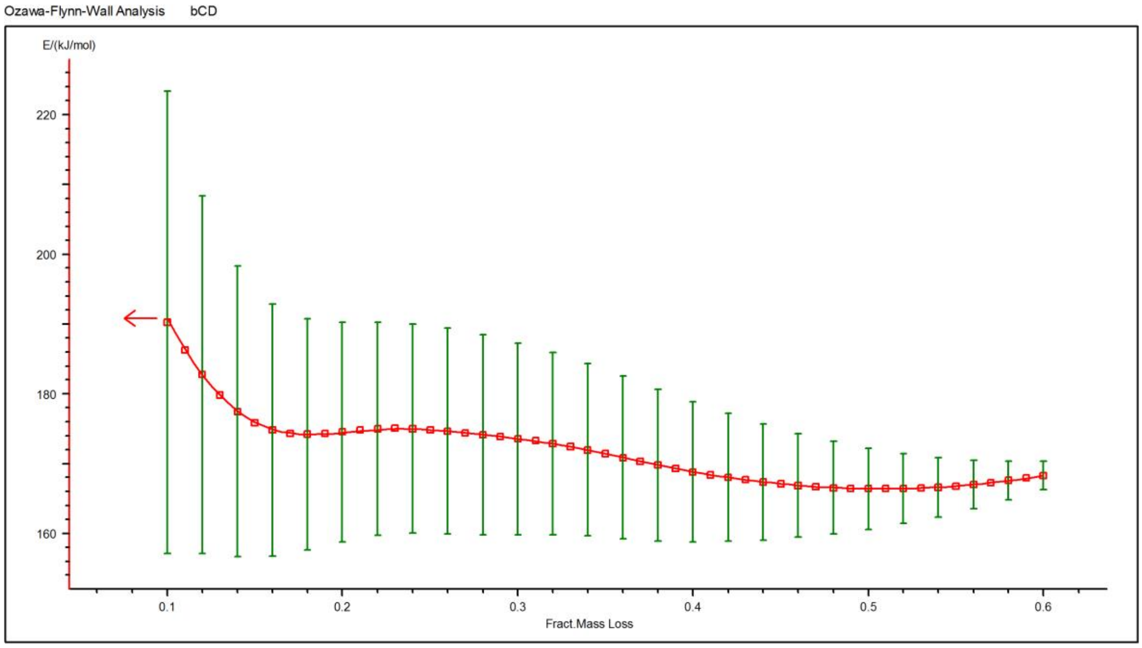

Figure S9. Isoconversional kinetic analysis (Flynn–Wall–Ozawa method) for beta-cyclodextrin thermal decomposition.

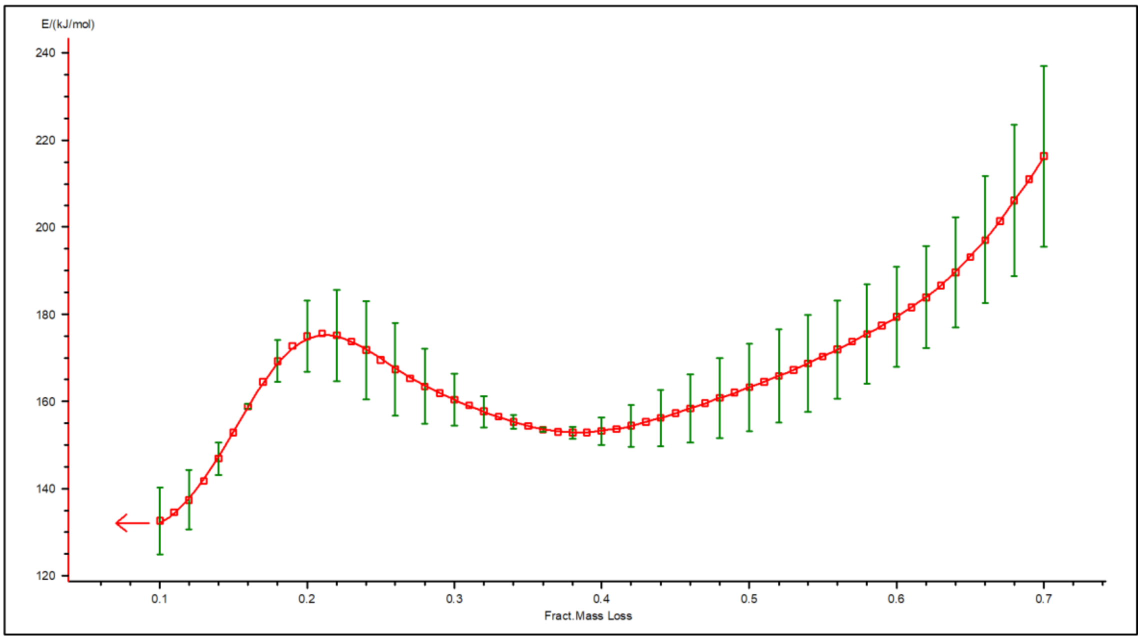

Figure S10. Isoconversional kinetic analysis (Friedman method) for beta-cyclodextrin thermal decomposition.

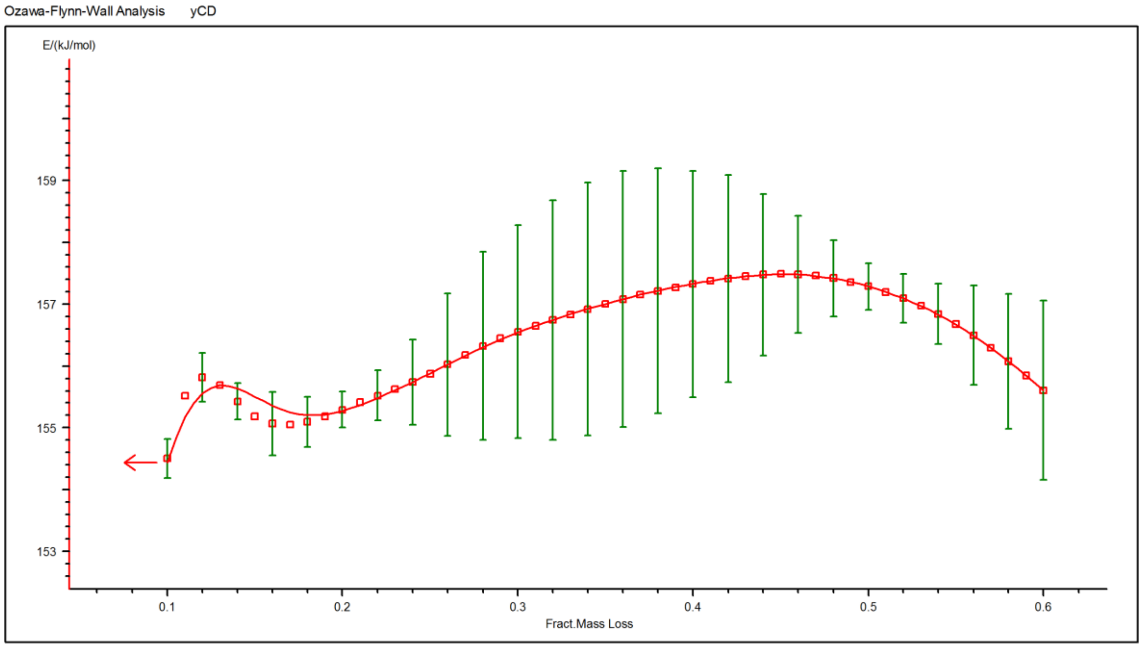

Figure S11. Isoconversional kinetic analysis (Flynn–Wall–Ozawa method) for gamma-cyclodextrin thermal decomposition.

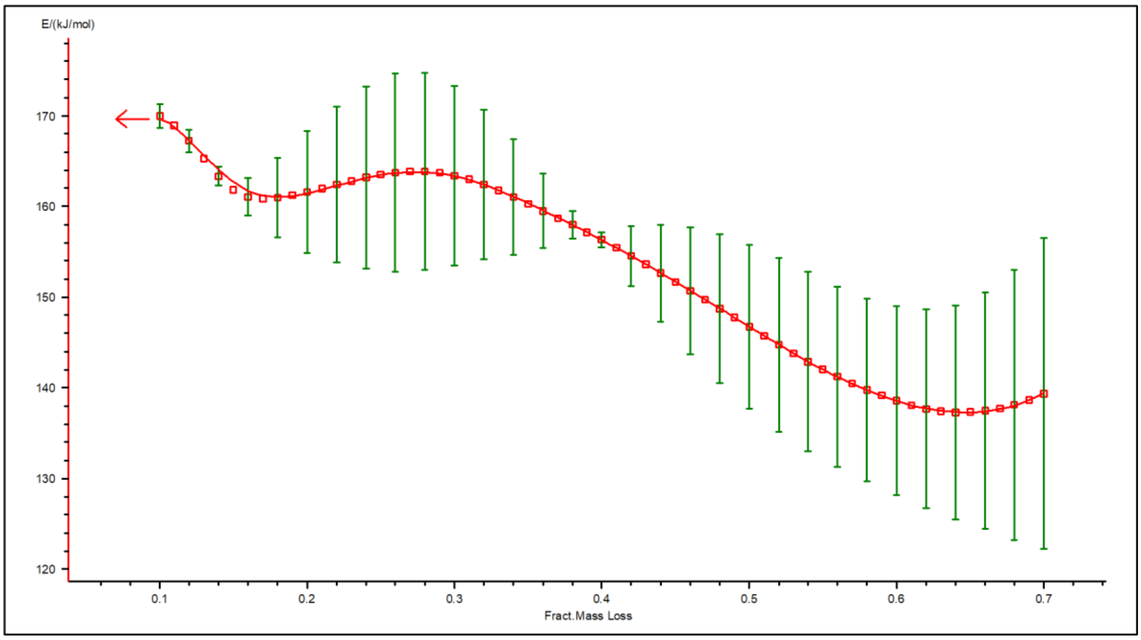

Figure S12. Isoconversional kinetic analysis (Friedman method) for gamma-cyclodextrin thermal decomposition.

Table S1. Activation energies (kJ mol^{-1}) of cyclodextrin thermal decomposition found by model-free methods. *

	alpha-cyclodextrin	beta-cyclodextrin	gamma-cyclodextrin
Flynn–Wall–Ozawa method	145.8	174.4	155.6
Friedman method	148.9	168.6	153.7

* averaged for conversion degree 0.1–0.7.

Thermokinetic analysis — best fit model

Table S2. Thermokinetics approximation for TG data of natural cyclodextrins decomposition by best model found.

	alpha-cyclodextrin	beta-cyclodextrin	gamma-cyclodextrin
best model	CnB	CnB	CnB
log A	9.036	12.989	9.937
E_a/kJ mol^{-1}	147	177	156
reaction order	3.01	3.37	2.77
correlation coefficient	0.999577	0.999321	0.999294

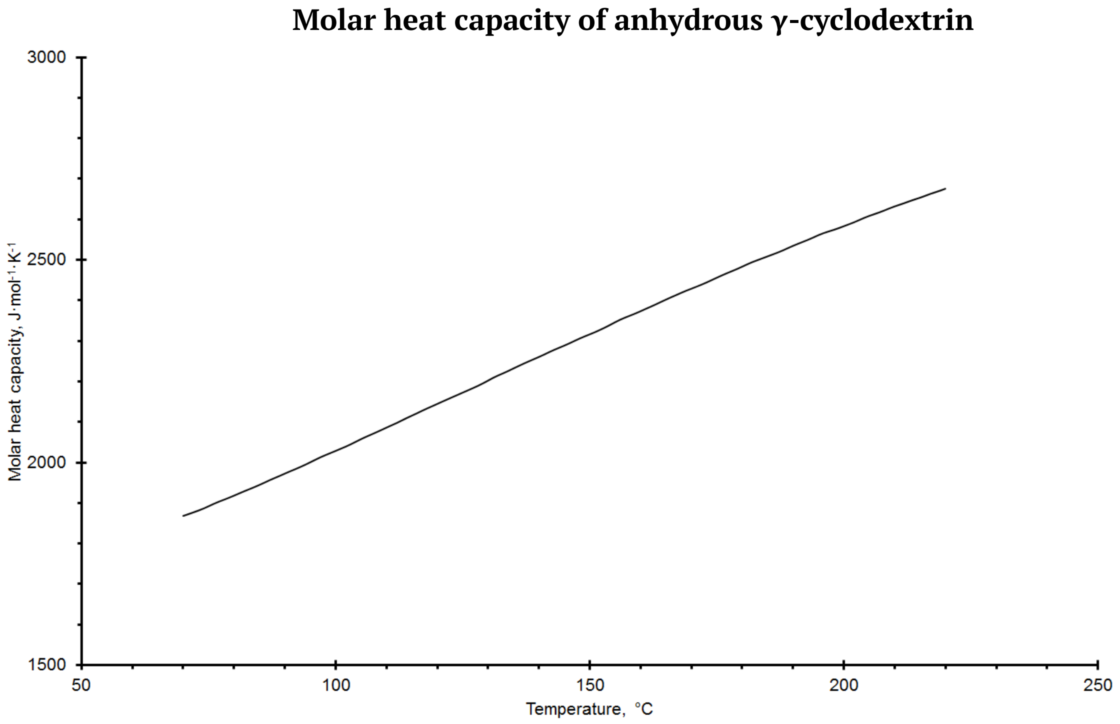

Figure S13. Molar heat capacity of anhydrous γ-cyclodextrin, obtained by DSC with usage of sapphire standard.

Table S3. Molar heat capacity of anhydrous γ-cyclodextrin, obtained by DSC with usage of sapphire standard.

Temp. /°C	C_p / J mol^{-1} K^{-1}	Temp. /°C	C_p / J mol^{-1} K^{-1}	Temp. /°C	C_p / J mol^{-1} K^{-1}
70	1868.65	120	2145.03	170	2429.13
71	1873.07	121	2150.69	171	2434.00
72	1877.41	122	2156.25	172	2439.13
73	1882.00	123	2161.77	173	2444.59
74	1886.99	124	2167.33	174	2450.41
75	1892.60	125	2172.79	175	2456.27
76	1898.44	126	2178.27	176	2461.91
77	1903.79	127	2183.86	177	2467.36
78	1908.68	128	2189.60	178	2472.52
79	1913.55	129	2195.74	179	2477.77
80	1918.78	130	2202.57	180	2483.50
81	1924.24	131	2209.35	181	2489.35
82	1929.58	132	2215.26	182	2494.71
83	1934.66	133	2220.72	183	2499.46
84	1939.80	134	2226.43	184	2504.05
85	1945.21	135	2232.47	185	2508.75
86	1950.94	136	2238.53	186	2513.52
87	1956.73	137	2244.37	187	2518.36
88	1962.24	138	2249.94	188	2523.55
89	1967.59	139	2255.36	189	2529.23
90	1973.07	140	2260.80	190	2534.74
91	1978.52	141	2266.53	191	2539.92
92	1983.82	142	2272.53	192	2544.95
93	1989.22	143	2278.26	193	2550.02
94	1994.98	144	2283.61	194	2555.47
95	2001.06	145	2288.80	195	2561.10
96	2007.28	146	2294.33	196	2566.09
97	2013.28	147	2300.36	197	2570.28
98	2018.72	148	2306.16	198	2574.18
99	2023.90	149	2311.51	199	2578.51
100	2029.18	150	2316.60	200	2583.22
101	2034.50	151	2321.81	201	2587.96
102	2039.82	152	2327.47	202	2592.88
103	2045.59	153	2333.40	203	2598.24
104	2051.80	154	2339.76	204	2603.76
105	2058.16	155	2346.53	205	2608.65
106	2064.03	156	2352.71	206	2612.95
107	2069.42	157	2358.10	207	2617.39
108	2074.99	158	2363.11	208	2622.27
109	2080.74	159	2368.17	209	2627.28
110	2086.54	160	2373.61	210	2631.99
111	2092.23	161	2379.24	211	2636.33
112	2097.88	162	2384.76	212	2640.61
113	2103.99	163	2390.47	213	2645.12
114	2110.23	164	2396.37	214	2649.43
115	2116.07	165	2402.25	215	2653.51
116	2121.89	166	2407.91	216	2658.10
117	2128.00	167	2413.37	217	2662.82
118	2133.86	168	2418.92	218	2666.97
119	2139.40	169	2424.24	219	2671.09
				220	2675.64

Data on γ-cyclodextrin melting

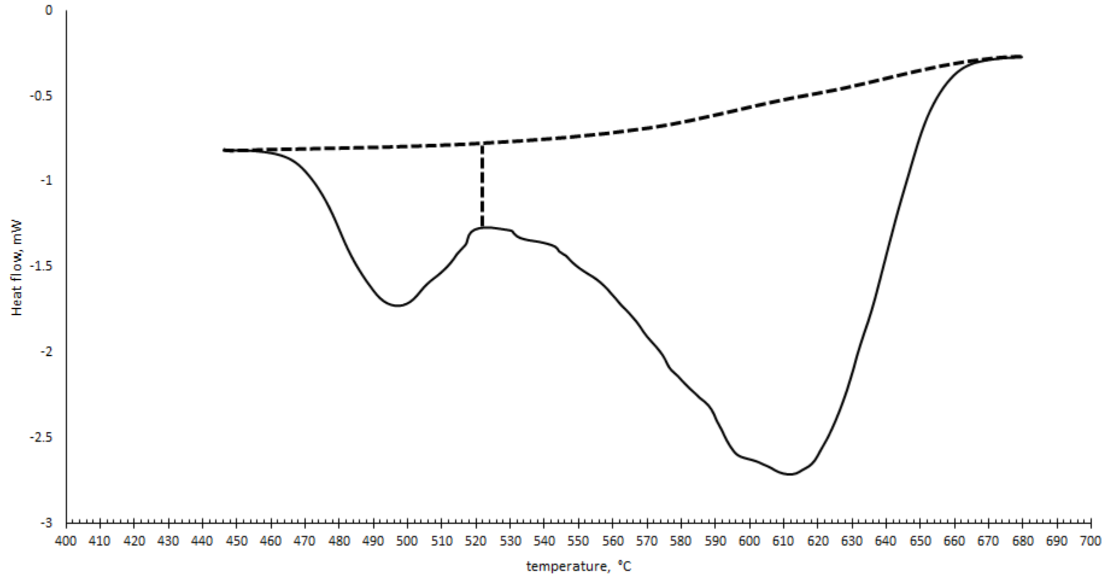

Figure S14. FSC curve of γ-cyclodextrin sample 1 with scanning rate of 10000 K·s^{-1}.

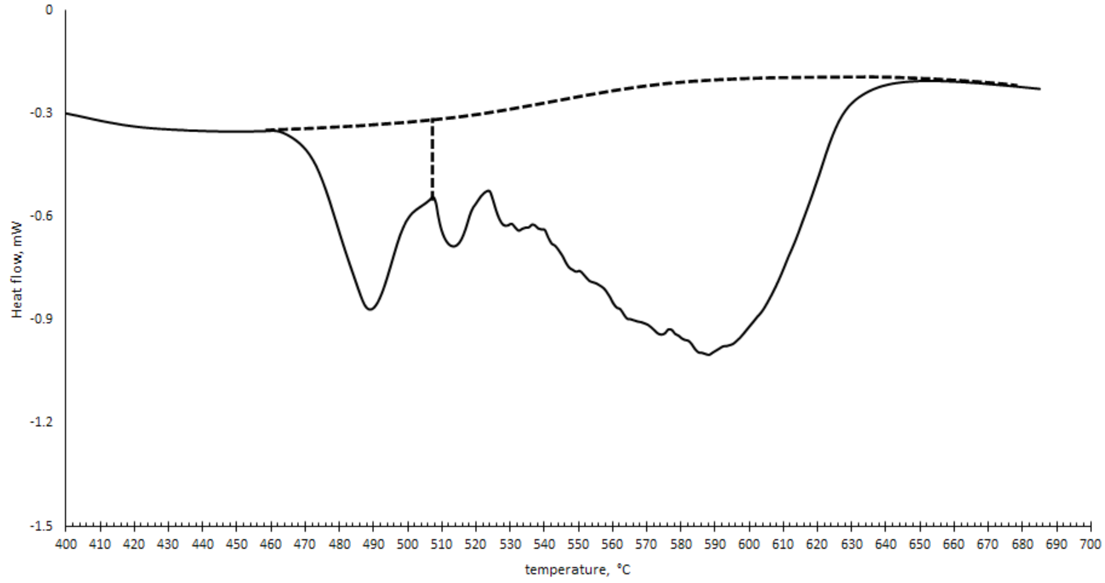

Figure S15. FSC curve of γ-cyclodextrin sample 2 with scanning rate of 10000 K·s^{-1}.

Table S4. Calculation of fusion enthalpy of γ-cyclodextrin.

	mol γ-CD, according to C_p measurements	Summary DSC integral of melting and decomposition /J	Peak fraction corresponding to melting	DSC integral of melting /J	Molar fusion enthalpy /kJ·mol^{-1}
sample 1	$1.5496 \cdot 10^{-11}$	$2.284 \cdot 10^{-5}$	0.1532	$3.498 \cdot 10^{-6}$	225.8
sample 2	$5.8643 \cdot 10^{-12}$	$7.012 \cdot 10^{-6}$	0.1814	$1.272 \cdot 10^{-6}$	216.9

References

1. Thomas, A.; Moinuddin, K.; Tretsiakova-McNally, S.; Joseph, P. A Kinetic Analysis of the Thermal Degradation Behaviours of Some Bio-Based Substrates. Polymers (Basel). 2020, 12, 1830, doi:10.3390/polym12081830.
